# Development strategy of non-GMO organism for increased hemoproteins in *Corynebacterium glutamicum*: a growth-acceleration-targeted evolution

**DOI:** 10.1007/s00449-024-02986-6

**Published:** 2024-03-18

**Authors:** Sehyeon Park, Seungki Lee, Taeyeon Kim, Ahyoung Choi, Soyeon Lee, Pil Kim

**Affiliations:** 1 Research Group of Novel Food Ingredients for Alternative Proteins, The Catholic University of Korea, Bucheon, Gyeonggi 14662 Republic of Korea; 2 Department of Biotechnology, The Catholic University of Korea, Bucheon, Gyeonggi 14662 Republic of Korea

**Keywords:** *Corynebacterium glutamicum*, Hemoproteins, Adaptive evolution, Positive feedback

## Abstract

Heme, found in hemoproteins, is a valuable source of iron, an essential mineral. The need for an alternative hemoprotein source has emerged due to the inherent risks of large-scale livestock farming and animal proteins. *Corynebacterium glutamicum*, regarded for Qualified Presumption of Safety or Generally Recognized as Safe, can biosynthesize hemoproteins. *C. glutamicum* single-cell protein (SCP) can be a valuable alternative hemoprotein for supplying heme iron without adversely affecting blood fat levels. We constructed the chemostat culture system to increase hemoprotein content in *C. glutamicum* SCP. Through adaptive evolution, hemoprotein levels could be naturally increased to address oxidative stress resulting from enhanced growth rate. In addition, we used several specific plasmids containing growth-accelerating genes and the *hemA* promoter to expedite the evolutionary process. Following chemostat culture for 15 days, the plasmid in selected descendants was cured. The evolved strains showed improved specific growth rates from 0.59 h^−1^ to 0.62 h^−1^, 20% enhanced resistance to oxidative stress, and increased heme concentration from 12.95 µg/g-DCW to 14.22–15.24 µg/g-DCW. Notably, the putative peptidyl-tRNA hydrolase-based evolved strain manifested the most significant increase (30%) of hemoproteins. This is the first report presenting the potential of a growth-acceleration-targeted evolution (GATE) strategy for developing non-GMO industrial strains with increased bio-product productivity.

**Supplementary Information:**

The online version contains supplementary material available at 10.1007/s00449-024-02986-6.

## Introduction

Iron helps prevent anemia, fetal development, oxygen transport, energy metabolism, muscle function, immune system, and the reduction of restless leg syndrome [1–4]. Iron is consumed as non-heme iron, present in animal and plant foods, and heme iron, found primarily in animal foods. The absorption of non-heme iron is influenced by various dietary components such as phytates, oxalates, polyphenols, and tannins, resulting in a variable bioavailability of approximately 1–10%. In contrast, heme iron absorption remains almost unaffected by dietary components, allowing for high bioavailability of up to 40% [5–9].

Heme iron is ingested in the form of hemoproteins, such as hemoglobin and myoglobin, found in animal foods. Red meat, especially from mammals such as cattle and pigs, is the richest source of hemoproteins. It also stimulates gastric acid production, promoting non-heme iron absorption [5–7]. However, animal proteins can harm blood fat levels and intestinal microbiota [10–16]. Large-scale livestock farming for red meat significantly contributes to global warming and environmental pollution, including soil, water, and air pollution. Therefore, it is a serious issue that must be addressed for reasons related to animal ethics and public health and for the continuation of humanity [17–24]. Considering the nutritional importance of heme iron and the challenges associated with its sourcing, various alternative hemoprotein sources should be developed for all individuals, including those who face barriers accessing meat or are vegetarian.

Leghemoglobin from nitrogen-fixing root nodules of leguminous plants is one of the alternative hemoproteins. However, direct extraction of leghemoglobin from roots demands substantial soil space for large-scale soybean cultivation. Furthermore, harvesting poses risks of soil erosion and the release of stored carbon. To overcome this, researchers conducted to express leghemoglobin in yeast through genetic modification, and Impossible Foods has finally succeeded in commercializing it [25]. Microbial-based single-cell proteins (SCP) can be promising replacement hemoprotein candidates [26–28]. *Corynebacterium glutamicum*, classified for Qualified Presumption of Safety (QPS) or Generally Recognized as Safe (GRAS) microorganism, can biosynthesize hemoproteins [29–31]. Previous studies have revealed that *C. glutamicum* heme-SCP has health benefits by positively influencing intestinal microbiota and blood fat levels in pet dog or obese mouse models [32, 33]. Hemoproteins in *C. glutamicum* contribute to microbial growth and resistance against oxidative stress. These proteins include cytochrome c oxidase, cytochrome P450, catalase, peroxidase, cytochrome bd oxidase, and nitrite reductase [34–36]. Thus, the hemoprotein synthesis level is correlated with the microbe’s growth rate and antioxidant capacity [37, 38]. Chemostat culture systems enable the selection of strains exhibiting improved growth rates through adaptive evolution [39].

In this study, we applied *C. glutamicum* carrying a specific genetic combinational plasmid to chemostat cultures. The plasmid in selected descendants was subsequently cured. Through a comparative analysis of oxidative stress resistance and heme concentration among the evolved strains, we evaluated the effect of evolution on increasing hemoprotein synthesis.

## Materials and methods

### Mouse study

#### Rearing and diet

Five-week-old male mice (C57BL/6N) were housed in the facility, where access to food and drink was not restricted, and the lighting was adjusted to 12 h each day (07:00 to 19:00). AIN93-G was used as a normal diet (ND). The *C. glutamicum* heme-SCP (5 g, dried biomass of hemoprotein-rich bacterial cells, purchased from Hemolab Ltd. Co., Seoul, South Korea) was blended with AIN93-G (1 kg) to prepare the ND with 0.05% *C. glutamicum* heme-SCP diet. The prepared diets (ND or ND with 0.05% *C. glutamicum* heme-SCP) were administered to each mouse group (*n* = 3) for 4 weeks. Throughout the test, body weight and intake of food and water were measured every 3 days.

#### Autopsy

Mice were anesthetized by CO_2_ gas, and blood samples were taken from the abdominal aorta. The blood sample was placed at 18 °C for 30 min and then centrifuged (1,200 × *g*, 15 min, 4 °C) to separate the serum. The serum samples were stored in a deep freezer until analyses. The levels of triglyceride (TG), total cholesterol (T-chol), high-density lipoprotein-cholesterol (HDL-c), and low-density lipoprotein-cholesterol (LDL-c) in the serum were measured using an autoanalyzer (model 7600II; Hitachi, Tokyo, Japan) in a biochemical institutional facility (Korea Non-clinic Test Support Center, Seongnam, South Korea). Data were expressed as the mean difference, 95% confidence interval (CI), and effect size. The effect size was calculated as Cohen’s *d* value; a small effect corresponds to Cohen’s *d* around 0.2. A medium effect corresponds to Cohen’s *d* around 0.5. A large effect corresponds to Cohen’s *d,* around 0.8 or higher.

### Bacterial strains, media, and batch culture

*Corynebacterium glutamicum* ATCC13032 (American Type Culture Collection) was used for the wild type (WT) of the experimental evolution. The MCGC minimal medium was used for all *C. glutamicum* culture [40]. All cases of *C. glutamicum* cultures were performed at 30 °C and 200 rpm. *C. glutamicum* stock cells were pre-cultured with 20 mL volume in a 100 mL baffled flask to optical density (OD) 1–2 at 600 nm. The main culture was incubated with 50 mL volume in a 250 mL bottom baffled flask (Duran GL45, Duran Inc., Germany). The initial OD_600_ of the main culture was adjusted to 0.1 using a pre-culture solution. Biomass was estimated by measuring OD_600_ and converting it into g-DCW (dry cell weight)/L unit by the coefficient of 0.3.

### Plasmids construction

Cloning and transformation followed the method described by Sambrook et al. [41]. using *Escherichia coli* DH10B (Invitrogen Inc., USA). The genomic DNA of *C. glutamicum* ATCC13032 was employed for the cloning of various genetic elements, including the *hemA* promoter (*PhemA*: 100 bp upstream from *hemA*), the gene encoding the iron-siderophore ABC transporter substrate-binding protein (SBP, GenBank: AUI01500.1), the cold-shock protein A (CspA, GenBank: AUH99802.1), the putative peptidyl-tRNA hydrolase (Pth, GenBank: AUI01932.1), and the helix-turn-helix transcriptional regulator (RamA, GenBank: AUI01965.1). Each coding region was amplified using the polymerase chain reaction (PCR) method, and PCR products were digested by restriction enzyme. The information of primer sequence and restriction enzyme are listed in Table S1. The promoter-probe shuttle vector pSK1Cat was used for cloning [42]. First, the *PhemA* fragment was inserted into the vector cut with *BamH*I and *Sal*I using T4 DNA ligase. Then, the SBP or *cspA* fragment was inserted into *PhemA*-pSK1Cat cut with *Sal*I and *Pst*I. The Pth or *ramA* fragment was inserted into *PhemA*-pSK1Cat cut only with *Sal*I (Fig. S1). The constructed plasmids were transformed into the *C. glutamicum* by electroporation [43]. Plasmids and strains are listed in Table S2.

### Chemostat culture for adaptive evolution

The chemostat culture system was established as follows: a 250 mL bottom baffled flask equipped with a screw cap (3-Port, For id. Φ3.2 mm Tubing), rigid PTFE tubing, and flexible silicon tubing for the inlet and outlet, and 0.2 µm PTFE membrane filter for airflow. The pre-culture solution was inoculated (initial OD_600_ = 0.1) without inlet/outlet feedings. After 5 h of the incubation, pumps for inlet feedings were started with a dilution rate of 0.55 h^−1^. The culture volume was maintained by extracting culture at an equivalent flow rate as the fresh medium feed. Feed rates were gradually increased throughout the continuous culture steps, taking care to avoid excessive dilution. This allows for the persistence of only the population with faster doubling times within the culture vessel. As each feeding reservoir was exhausted, it was replaced with a new reservoir in aseptic conditions.

### Plasmid curing

The pre-culture solution of the plasmid harboring strain was inoculated into MCGC medium containing 0.003% sodium dodecyl sulfate and incubated at 37 °C and 200 rpm for 24 h. Dilute the culture medium appropriately, spread it on MCGC agar medium, and culture it at 30 °C to obtain a single colony. Then, spiked on MCGC agar medium and MCGC agar medium containing kanamycin, and culture it at 30 °C to screen for plasmid cured colony.

### Analytical method

Cells were harvested to have an OD of approximately 40 in a screw-capped tube by centrifugation (10,000×*g* at 4 °C for 5 min). The cell pellet was washed once with phosphate-buffered saline (PBS; 1x, pH 7.4). The washed cell pellet was used for subsequent analysis.

#### Oxidative stress resistance test

Oxidative stress resistance was determined by measuring residual intracellular reactive oxygen species (ROS) using 2′,7′-dichlorofluorescein diacetate (DCFH-DA). DCFH-DA stock solution was prepared by dissolving it at 10 mM in dimethyl Sulfoxide. The stock solution was diluted to 10 µM in PBS. Add 1 mL of 10 µM DCFH-DA solution to the washed pellet and suspend. Incubate the suspension at 30 °C and 200 rpm for 1 h to facilitate the conversion of DCFH-DA into its fluorescent DCF form in the presence of ROS. After the incubation, cell pellets were collected by centrifugation (10,000×*g* at 4 °C for 5 min). The cell pellet was washed once with PBS to remove any extracellular DCFH-DA and suspended at 1 mL of PBS. The DCF fluorescence signal was measured using fluorescence spectrophotometry (Synergy Mx, BioTek, USA) with excitation at 485 nm and emission at 530 nm, using a 96-well plate.

#### Heme measurement

Heme was unbound and extracted from the bacterial hemoproteins using modified actone:HCl extraction methods [44]. The washed bacterial cell pellet was mixed with 1 mL of acetone:HCl (80:20) and 0.2 g of glass beads (212–300 µm), followed by homogenization for 30 s using a bead beater (FastPrep-24™ 5G bead beating system, MP Biomedicals, USA). After resting for 1 min, homogenization was repeated five more times. Subsequently, the lysate was incubated at − 20 °C for 20 min. Cell debris in lysate was removed by centrifugation (17,000×*g*, at 4 °C for 10 min), and the supernatant was used for measuring intracellular heme.

Heme amount was determined by measuring UV-absorbance using high-performance liquid chromatography (HPLC Agilent 1100) equipped with reverse-phase C18 column (C18, 3.5 μm, 150 mm × 4.6 mm, SunFire™). The temperature of the column oven was set at 40 °C. The mobile phase was composed of solvent A (methanol:acetonitrile = 10:90) and B (0.5% (v/v) trifluoroacetic acid in HPLC grade water). A linear gradient method (20–95%) of solvent A was applied for 0 to 7 min. The flow rate was maintained at 1 mL/min for a total analysis time of 10 min. The chromatogram peak of heme at about 6.7 min retention time was detected at a wavelength of 398 nm. The calibration curve was constructed using (0.01–1.5) µM hemin solution in acetone:HCl (80:20).

## Results and discussion

### Hemoprotein in *C. glutamicum*

While hemoprotein-rich red meat should be consumed for optimal iron intake [5–7], animal protein carries the inherent risk of increasing blood fat levels [10–13]. However, *C. glutamicum* heme-SCP supplementation did not adversely affect blood fat levels in the mice cohort of this study. Compared to fed ND only, supplementation with 0.05% *C. glutamicum* heme-SCP reduced blood TG (− 0.03 mmol/L, 95% CI − 0.056, − 0.005), T-Chol (− 0.328 mmol/L, 95% CI − 0.513, − 0.142), HDL-c (− 0.101 mmol/L; 95% CI − 0.11, − 0.091), and LDL-c (− 0.023 mmol/L; 95% CI − 0.037, − 0.008). Notably, reductions in T-chol and LDL-c showed large effect sizes with absolute *d* values of over 0.8 (Table 1). Although we could not make a conclusive discussion due to the small number of replicates (*n* = 3), the outcomes correspond with the previous study [33]. Therefore, *C. glutamicum* heme-SCP can be a valuable alternative hemoprotein for supplying heme iron.

**Table 1.** Comparison of TG, T-Chol, HDL-c, and LDL-c in a mouse model (*n *= 3) depending on diet

	ND only	ND with 0.05% C. *glutamicum *heme-SCP	Mean difference (95% CI)	Cohen’s *d*
TG	0.81 ± 0.12	0.78 ± 0.10	− 0.03 (− 0.056, − 0.005)	− 0.276
I-Chol	1.89 ± 0.48	1.56 ± 0.31	− 0.328 (− 0.513, − 0.142)	− 0.833
HDL	1.09 ± 0.19	0.99 ± 0.18	− 0.101 (− 0.11, − 0.091)	− 0.535
LDL-c	0.11 ± 0.03	0.09 ± 0.02	− 0.023 (− 0.037, − 0.008)	− 0.883

**Fig. 1:**
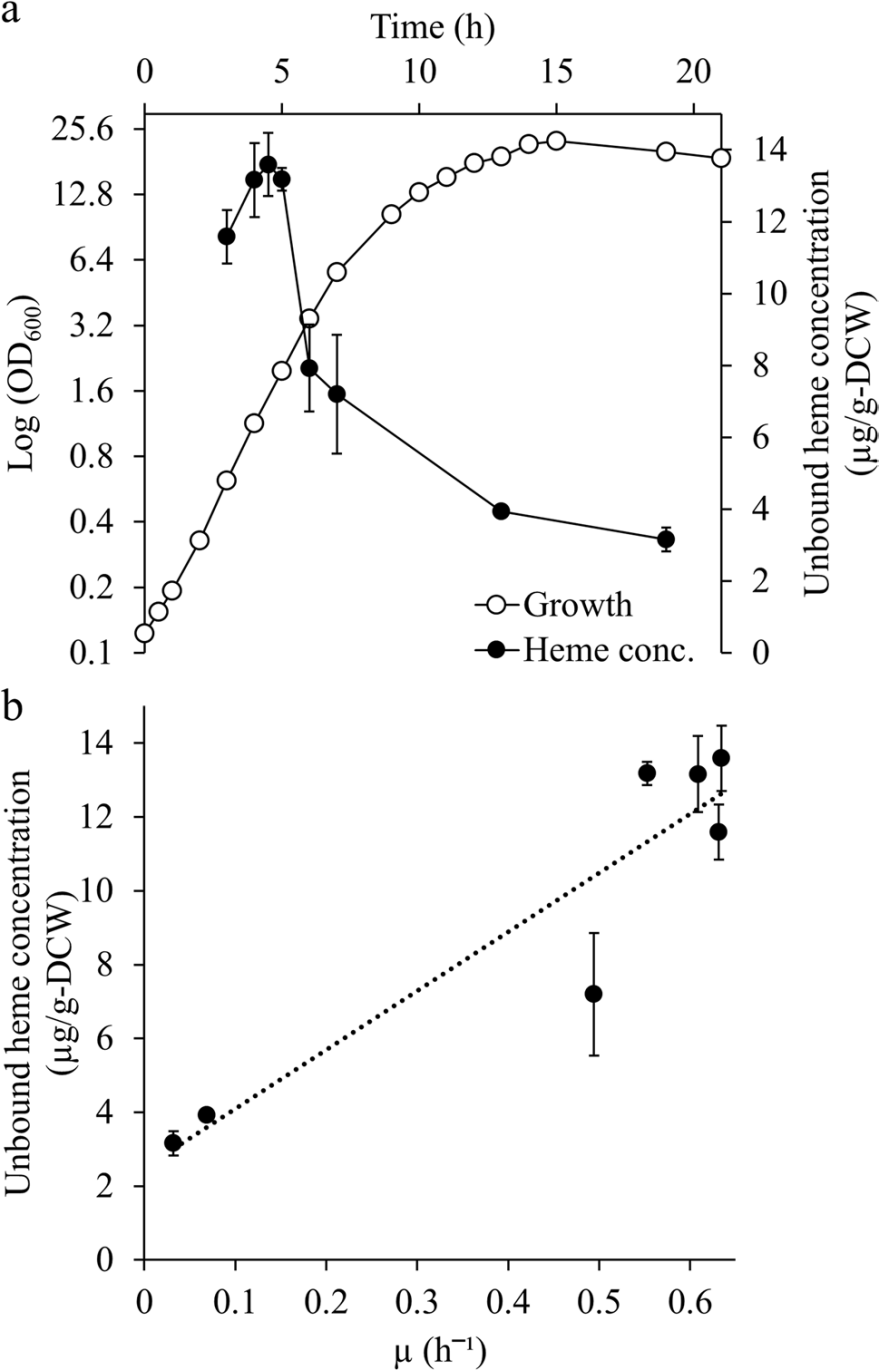
**a** Growth curves and heme concentrations of *C. glutamicum*. **b** Heme concentration of *C. glutamicum* depends on the specific growth rate (µ). Open circles indicate log (OD_600_), and closed circles indicate heme concentration

Increasing the hemoprotein content per cell is essential to enhance the value of *C. glutamicum* heme-SCP as an alternative hemoprotein source. Hemoproteins in *C. glutamicum* are associated with both growth rate and oxidative stress management, suggesting that their levels could vary depending on the microbial growth state [34–38]. The heme concentration in *C. glutamicum* peaked during the mid-log phase at 4–5 h (Fig. 1a). Bacterial cells within the mid-log phase experience severe oxidative stress due to a near-maximal specific growth rate arising from abundant nutrients in the medium. Therefore, the cause of the peak heme concentration can be attributed to the high hemoprotein levels [34–38]. In addition, a positive correlation was observed between the instantaneous specific growth rate and heme concentrations throughout the entire growth phase (Fig. 1b). Consequently, higher concentrations of hemoproteins can be obtained from cells with a faster specific growth rate.

### Positive feedback and growth-acceleration-targeted evolution (GATE)

**Fig. 2:**
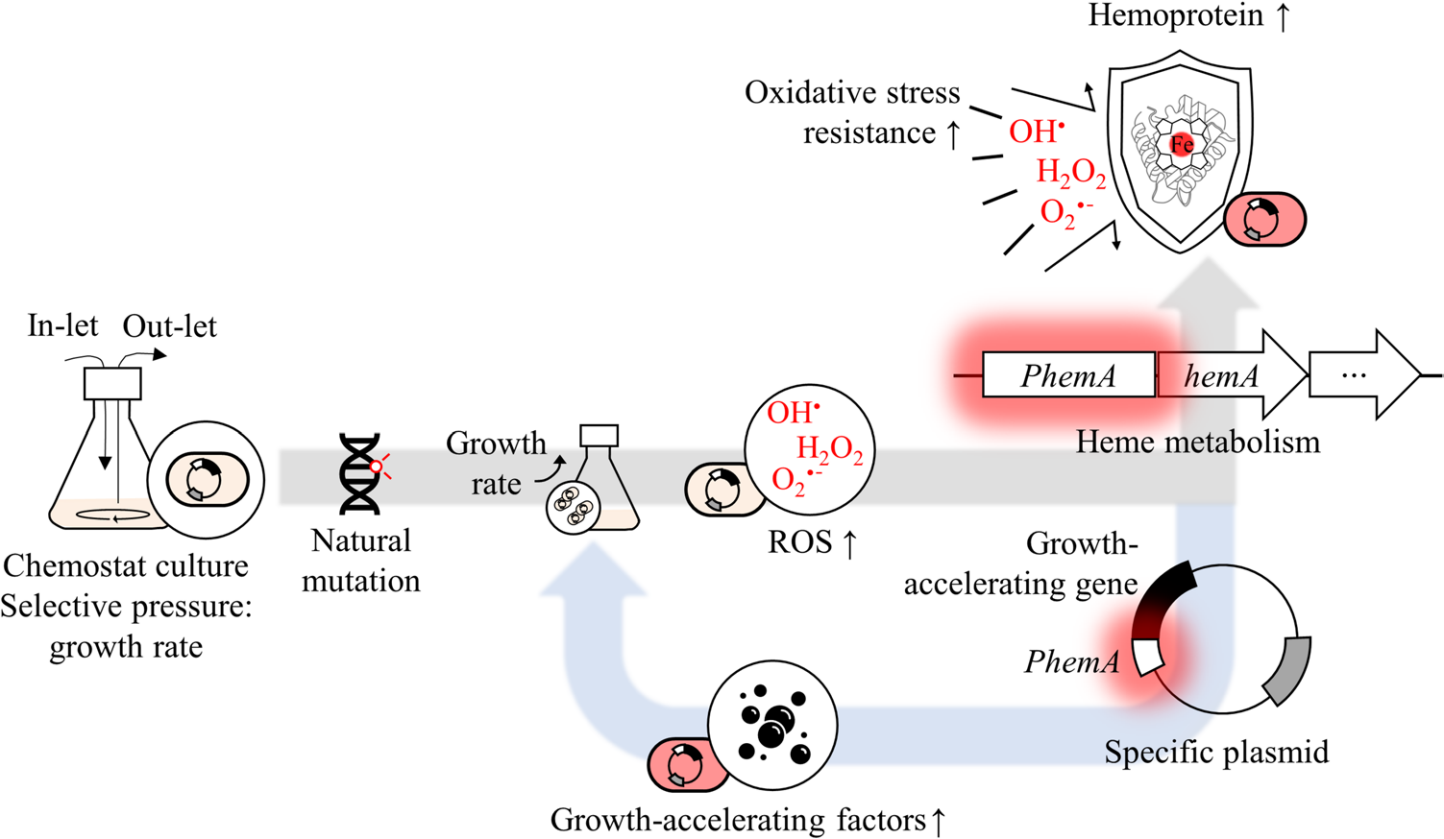
Schematic diagram of GATE. The red neon sign means activation of *PhemA*. The gray arrow indicates the one direction of normal evolution. The blue arrow is a positive feedback loop formed by the specific plasmid is present

Chemostat culture, established with a progressively increasing media replacement rate, can select evolved strains with improved specific growth rates through adaptive evolution [39]. To enhance the effect of evolution, we introduced the concept of GATE. A specific genetic combinational plasmid was employed for improving hemoprotein levels in the evolutionary process. We constructed *PhemA*-SBP, *PhemA*-*cspA*, *PhemA*-Pth, and *PhemA*-*ramA* by combining genes encoding proteins that accelerate the specific growth rate and the *PhemA* (Fig. S1). Expression products of growth-accelerating genes (SBP, CspA, Pth, RamA) have been verified to accelerate growth rates in previous studies [39, 45]. *PhemA*, serving as the trigger for these genes, originated from the *hemA* promoter. The expression product of *hemA* is an enzyme with the highest Gibbs free energy. It may also be a bottleneck because it catalyzes the initial step of the heme synthesis pathway [31]. Therefore, amplification of *PhemA* activation can be crucial for increasing hemoprotein production. When these plasmids are transformed into a *C. glutamicum*, a positive feedback loop could be formed in which an increase in hemoprotein also enhances the growth rate (Fig. 2). This mechanism could provide a competitive advantage in adaptive evolution, facilitating the natural selection of mutants showing increased hemoprotein levels.

**Fig. 3:**
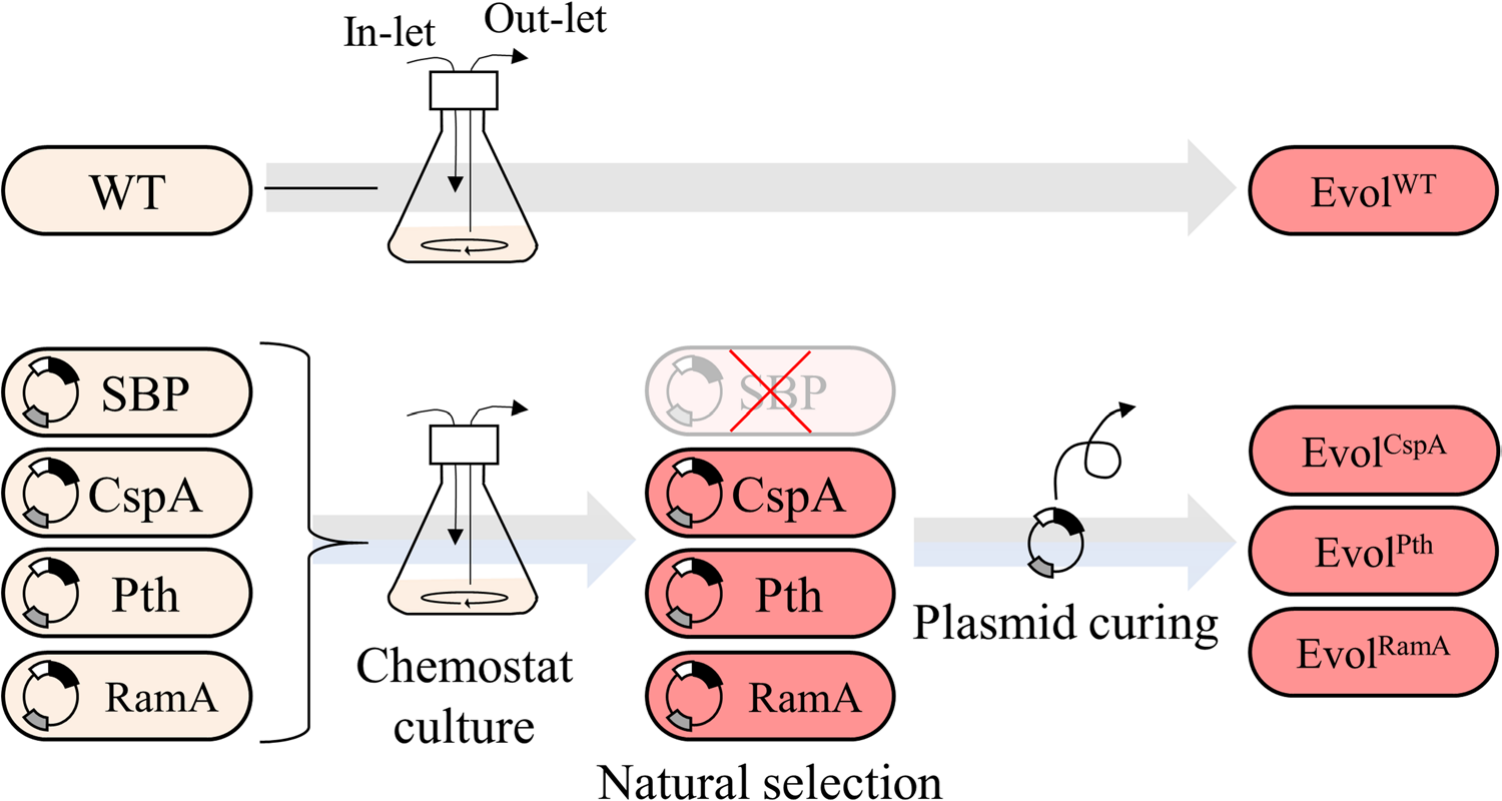
Schematic diagram of chemostat culture and evolution selection. The chemostat culture was conducted for 15 days. The *PhemA*-SBP-containing strain was eliminated. After plasmid curing, Evol^CspA^, Evol^Pth^, and Evol^RamA^ were selected

Since the functional mechanisms of SBP, CspA, Pth, and RamA are different [39, 45], the degree of growth acceleration by positive feedback will vary. Thus, we initiated a growth competition among strains within the same culture to readily select evolved ones showing the most enhanced growth rates. Following a 15-day chemostat culture, strains *PhemA*-*cspA*, *PhemA*-Pth, and *PhemA*-*ramA* survived, but the strain containing *PhemA*-SBP was eliminated (Fig. 3). Elimination of the *PhemA*-SBP-containing strain was probably due to an excessive iron uptake leading to intractable oxidative stress levels [45–48]. Plasmids of selected strains were cured. The selected evolutionary strains have emerged from natural mutations without artificial genetic manipulation. Therefore, upon confirmation that the plasmid did not affect the genomic DNA, Evol^CspA^, Evol^Pth^, and Evol^RamA^ hold the potential to be classified as a non-genetically modified organism (non-GMO).

### Influence of GATE on hemoprotein content

**Fig. 4:**
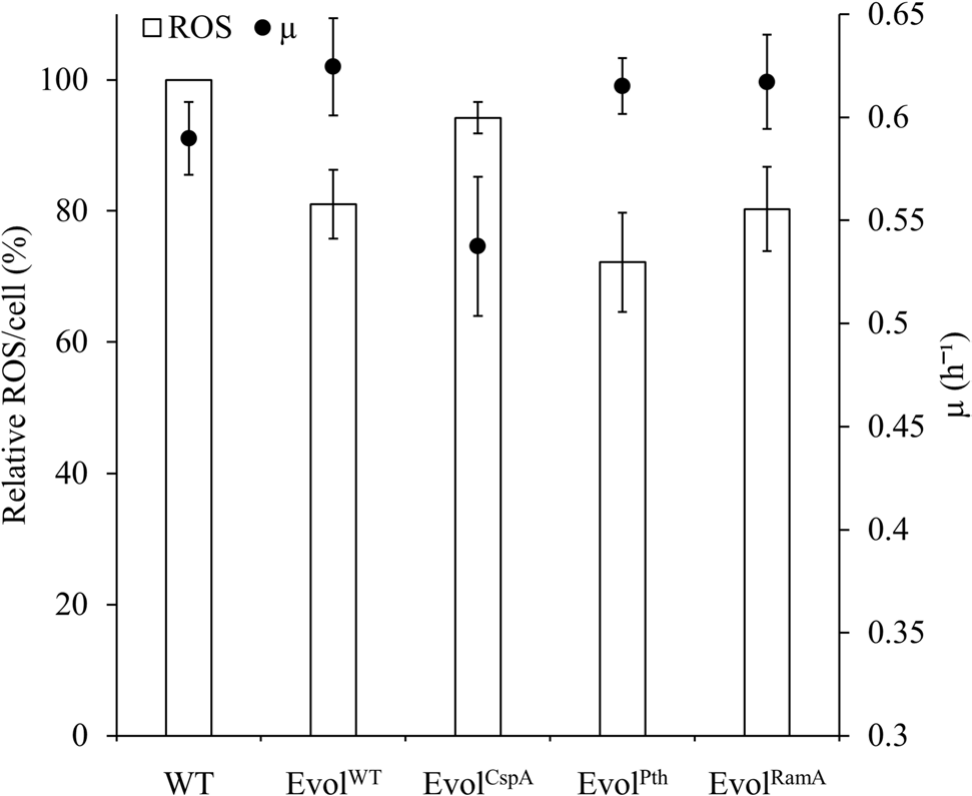
Assessment of oxidative stress resistance and specific growth rate (µ) at the mid-log phase (4–5 h of culture). Resistance to oxidative stress was displayed as intracellular ROS levels. White bars indicate relative ROS levels, and closed circles indicate µ

**Fig. 5:**
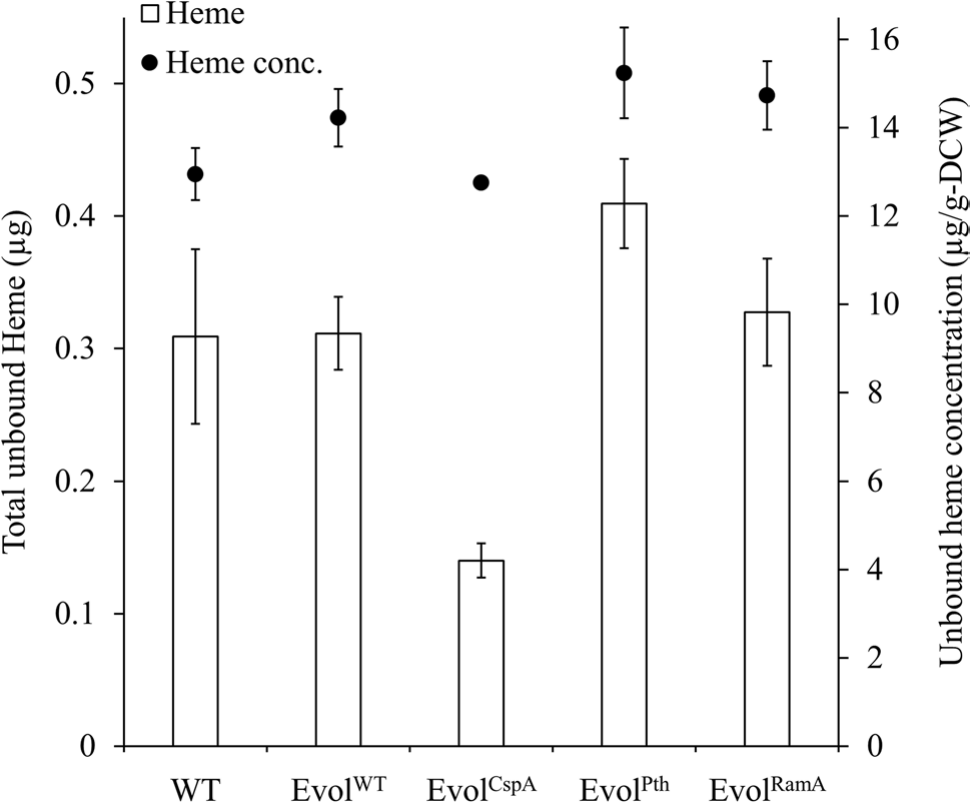
Measurement of intracellular heme amount and concentration at the mid-log phase (4–5 h of culture). White bars indicate heme amount, and closed circles indicate heme concentration

Since the evolution trajectory of evolved strains probably varied due to their prior possessed plasmids, hemoprotein levels could be different. To evaluate the genetic combination that facilitated the most effective evolution in improving hemoprotein levels, we compared the specific growth rate, oxidative stress resistance, heme synthesis amount, and heme concentration among the evolved strains. In the mid-log phase (4–5 h of culture), the ROS level in Evol^CspA^ was not significantly different from that in WT, and the specific growth rate decreased. On the other hand, the ROS levels in Evol^WT^, Evol^Pth^, and Evol^RamA^ were approximately 20% lower than that in WT, accompanied by an increase in the specific growth rate from 0.59 h^−1^–0.62 h^−1^ (Fig. 4). This is presumed to be an adaptation to oxidative stress induced by the increased growth rate, leading to an improved ability to manage ROS. The heme concentration of EvolCspA was similar to the WT. However, Evol^WT^, Evol^CspA^, and Evol^RamA^ showed increased heme concentrations from 12.95 µg/g-DCW to 14.22–15.24 µg/g-DCW (Fig. 5). Considering the tendency of enhanced resistance to oxidative stress within these strains (Fig. 4), this demonstrates an increase in hemoprotein content. In particular, Evol^Pth^ showed the highest heme concentration, with heme synthesis amount showing a distinct increase (30%) relative to the WT strain (Fig. 5).

We presumed that the functional mechanism, rather than the performance of the growth-accelerating factor, significantly influenced the enhancement of hemoprotein levels during evolution. RamA is a global regulator crucial for governing acetate metabolism, glycolysis, TCA cycle, anaplerosis, gluconeogenesis, and glucose uptake [49]. Thus, pathways unrelated to growth acceleration may occur in positive feedback loops. Cold shock proteins have multiple functions, including transcriptional and translational regulation, adaptation to cold conditions, RNA chaperone activity, cell growth, and response to oxidative stress [50, 51]. These could bring about CspA-dependent oxidative stress resistance, in addition to the potential of inducing unintended pathways in the positive feedback loop. Thus, it is speculated that the results observed in Evol^CspA^ are caused by evolution independent of hemoprotein. Meanwhile, considering that the function of Pth is limited to the translation support part [52, 53], the positive feedback probably circulates without substantial variables. Therefore, the *PhemA-*Pth combination is the most effective tool for GATE to improve hemoprotein levels.

## Conclusion

*Corynebacterium glutamicum* heme-SCP displayed the potential as an alternative hemoprotein source with a favorable effect on blood fat levels. *C. glutamicum* SCP with a high hemoprotein concentration can be obtained from cells with a faster growth rate. A plasmid combining *PhemA* and a growth-accelerating gene could form positive feedback in which an increase in hemoprotein also enhances the growth during adaptive evolution. Notably, the *PhemA*-Pth combination proved most effective in improving hemoprotein levels. We presumed that the effective positive feedback was attributed to the limited function of Pth. Insights into the positive feedback mechanisms will aid adaptive evolution for increasing bioproducts associated with growth. In conclusion, we propose that the GATE is a potent strategy for developing non-GMO industrial strains with increased bio-productivity.

### Supplementary Information

Below is the link to the electronic supplementary material.

Supplementary file1 (XML 0 kb)

## Data Availability

Raw data are available on request.
