# Supplementary material for: Development strategy of non-GMO organism for increased hemoproteins in Corynebacterium glutamicum: a growth-acceleration-targeted evolution
Source: Bioprocess Biosyst Eng. 2024 Mar 18;47(4):549–56. doi: 10.1007/s00449-024-02986-6 (PMC11003892; doi:10.1007/s00449-024-02986-6)
Supplement: Supplementary file 1 — Supplementary file1 (XML 0 kb) [file 449_2024_2986_MOESM1_ESM.pptx]

## Slide 1
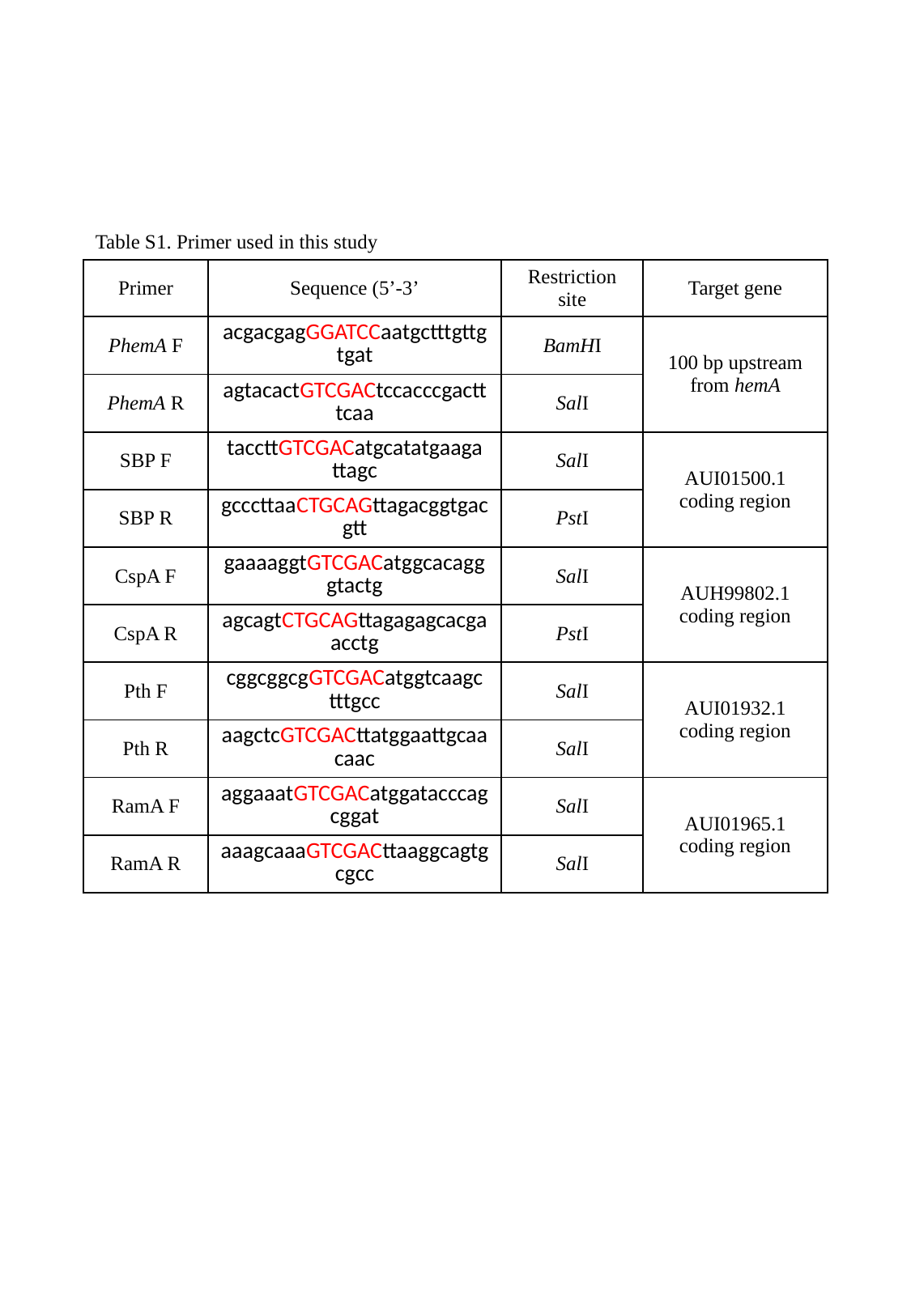

Table S1. Primer used in this study
| Primer | Sequence (5’-3’ | Restriction site | Target gene |
| --- | --- | --- | --- |
| PhemA F | acgacgagGGATCCaatgctttgttgtgat | BamHI | 100 bp upstream from hemA |
| PhemA R | agtacactGTCGACtccacccgactttcaa | SalI | |
| SBP F | taccttGTCGACatgcatatgaagattagc | SalI | AUI01500.1 coding region |
| SBP R | gcccttaaCTGCAGttagacggtgacgtt | PstI | |
| CspA F | gaaaaggtGTCGACatggcacagggtactg | SalI | AUH99802.1 coding region |
| CspA R | agcagtCTGCAGttagagagcacgaacctg | PstI | |
| Pth F | cggcggcgGTCGACatggtcaagctttgcc | SalI | AUI01932.1 coding region |
| Pth R | aagctcGTCGACttatggaattgcaacaac | SalI | |
| RamA F | aggaaatGTCGACatggatacccagcggat | SalI | AUI01965.1 coding region |
| RamA R | aaagcaaaGTCGACttaaggcagtgcgcc | SalI | |

## Slide 2
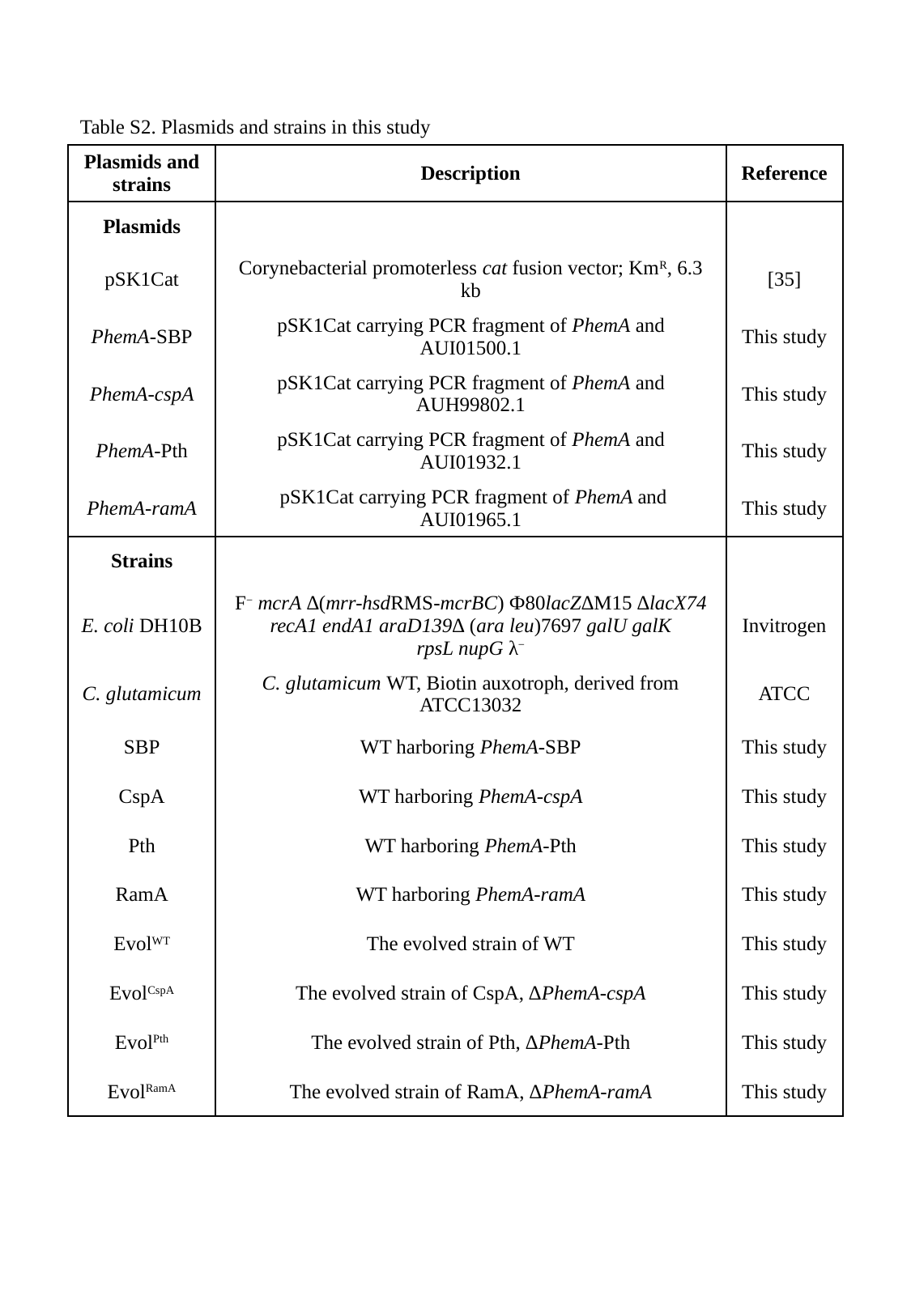

Table S2. Plasmids and strains in this study
| Plasmids and strains | Description | Reference |
| --- | --- | --- |
| Plasmids | | |
| pSK1Cat | Corynebacterial promoterless cat fusion vector; KmR, 6.3 kb | [35] |
| PhemA-SBP | pSK1Cat carrying PCR fragment of PhemA and AUI01500.1 | This study |
| PhemA-cspA | pSK1Cat carrying PCR fragment of PhemA and AUH99802.1 | This study |
| PhemA-Pth | pSK1Cat carrying PCR fragment of PhemA and AUI01932.1 | This study |
| PhemA-ramA | pSK1Cat carrying PCR fragment of PhemA and AUI01965.1 | This study |
| Strains | | |
| E. coli DH10B | F− mcrA ∆(mrr-hsdRMS-mcrBC) Ф80lacZ∆M15 ∆lacX74 recA1 endA1 araD139∆ (ara leu)7697 galU galK rpsL nupG λ− | Invitrogen |
| C. glutamicum | C. glutamicum WT, Biotin auxotroph, derived from ATCC13032 | ATCC |
| SBP | WT harboring PhemA-SBP | This study |
| CspA | WT harboring PhemA-cspA | This study |
| Pth | WT harboring PhemA-Pth | This study |
| RamA | WT harboring PhemA-ramA | This study |
| EvolWT | The evolved strain of WT | This study |
| EvolCspA | The evolved strain of CspA, ∆PhemA-cspA | This study |
| EvolPth | The evolved strain of Pth, ∆PhemA-Pth | This study |
| EvolRamA | The evolved strain of RamA, ∆PhemA-ramA | This study |

## Slide 3
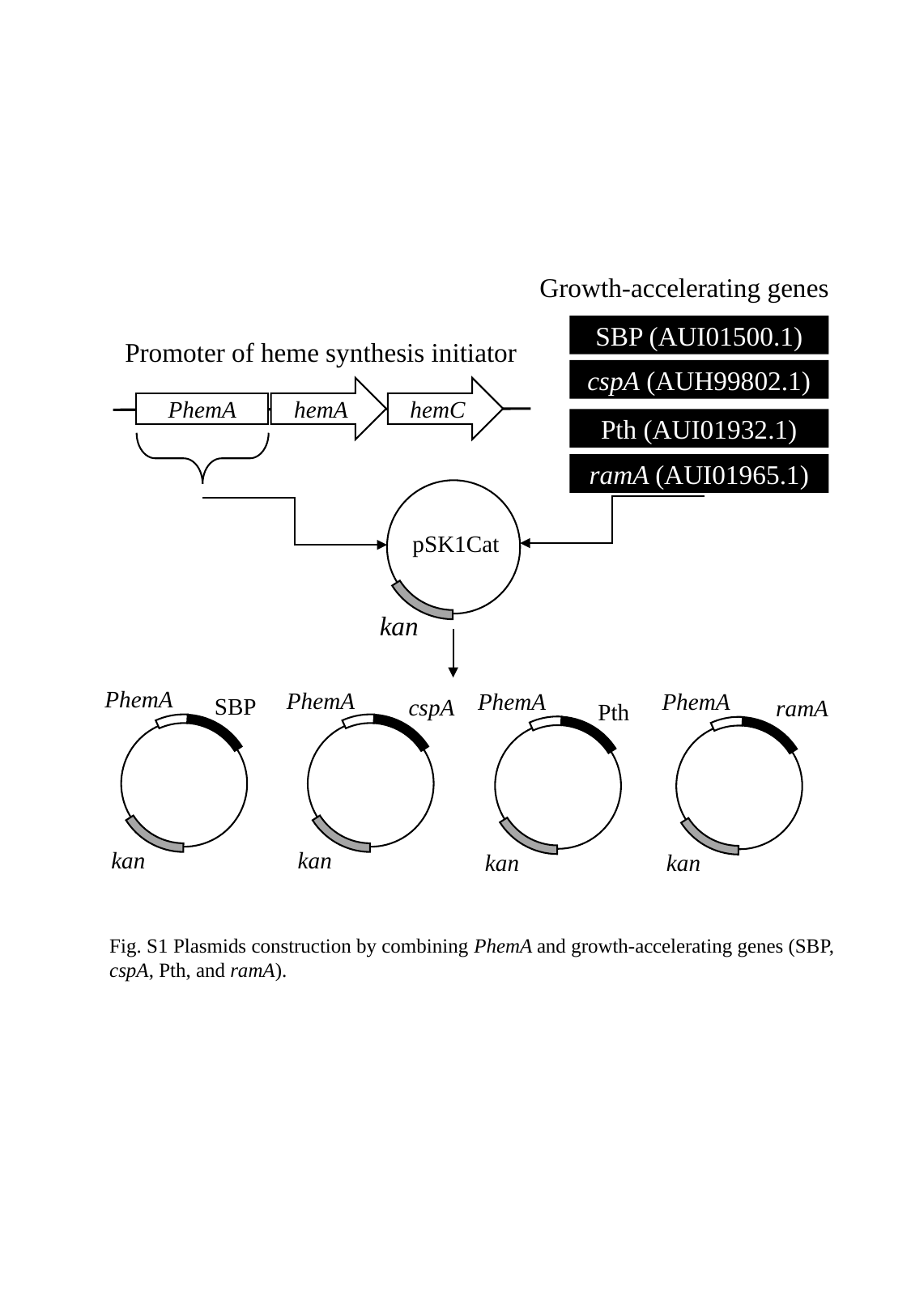

Growth-accelerating genes
SBP (AUI01500.1)
cspA (AUH99802.1)
Pth (AUI01932.1)
ramA (AUI01965.1)
Promoter of heme synthesis initiator
hemA
hemC
PhemA
pSK1Cat
kan
PhemA
SBP
kan
PhemA
cspA
kan
PhemA
Pth
kan
PhemA
ramA
kan
Fig. S1 Plasmids construction by combining PhemA and growth-accelerating genes (SBP, cspA, Pth, and ramA).
